# Relationships between Meiofaunal Biodiversity and Prokaryotic Heterotrophic Production in Different Tropical Habitats and Oceanic Regions

**DOI:** 10.1371/journal.pone.0091056

**Published:** 2014-03-06

**Authors:** Antonio Pusceddu, Cristina Gambi, Cinzia Corinaldesi, Mariaspina Scopa, Roberto Danovaro

**Affiliations:** 1 Dipartimento di Scienze della Vita e dell’Ambiente, Università Politecnica delle Marche, Ancona, Italy; 2 Istituto Zooprofilattico Sperimentale dell’Abruzzo e del Molise ‘G. Caporale’, Experimental Regional Centre for Fishery and Acquacolture, Termoli, Italy; 3 Stazione Zoologica Anton Dohrn, Napoli, Italy; University of Sydney, Australia

## Abstract

Tropical marine ecosystems are among the most diverse of the world oceans, so that assessing the linkages between biodiversity and ecosystem functions (BEF) is a crucial step to predict consequences of biodiversity loss. Most BEF studies in marine ecosystems have been carried out on macrobenthic diversity, whereas the influence of the meiofauna on ecosystem functioning has received much less attention. We compared meiofaunal and nematode biodiversity and prokaryotic heterotrophic production across seagrass, mangrove and reef sediments in the Caribbean, Celebes and Red Seas. For all variables we report the presence of differences among habitats within the same region, and among regions within the same habitat. In all regions, the richness of meiofaunal taxa in reef and seagrass sediments is higher than in mangrove sediments. The sediments of the Celebes Sea show the highest meiofaunal biodiversity. The composition of meiofaunal assemblages varies significantly among habitats in the same region. The nematode beta diversity among habitats within the same region is higher than the beta diversity among regions. Although one site per habitat was considered in each region, these results suggest that the composition of meiofaunal assemblages varies primarily among biogeographic regions, whereas the composition of nematode assemblages varies more considerably among habitats. Meiofauna and nematode biodiversity and prokaryotic heterotrophic production, even after the removal of covariate effects linked with longitude and the quantity and nutritional quality of organic matter, are positively and linearly linked both across regions and within each habitat type. Our results confirm that meiofauna and nematode biodiversity may influence benthic prokaryotic activity, which, in turn, implies that diversity loss could have negative impacts on ecosystem functioning in these systems.

## Introduction

Marine coastal ecosystems provide crucially important goods and services to the human beings [1], [2]. Marine coastal ecosystems are characterized by the presence of a complex and heterogeneous mosaic of habitats, such as sandy beaches, rocky shores, mangrove forests, seagrass meadows, coral reefs and transitional zones linking terrestrial and coastal ecosystems. Nevertheless, because of their proximity with the planet region most dense in human population, the coastal oceans are exposed to multiple anthropogenic stressors, including, among the others, aquaculture, dredging, mining, pollution, species invasion, over-harvesting and destructive fishing practices, large-scale oil and gas operations, watershed and offshore renewable energy development, coastal engineering habitat having strong and direct effects on the marine biodiversity [3], [4].

The annual loss rates of five of the most important biogenic marine habitats (seagrass beds, salt marshes, coral and oyster reefs and mangrove forests) range between 1 and 9% [5]. Based on historical evidence, the total global loss of these habitats ranges from ca. 19% for coral reefs (in between 2004–2008) [6], to 29% (since 1879) for seagrass [7], to 35% (since 80ies) for mangrove forests [8] to >85% (in the last 20–130 years) for oyster reefs [9]. Many of these habitats, which are also hot spots of biodiversity, belong to tropical regions.

Ecological theory predicts that biodiversity can control ecosystems’ functioning, although outputs of correlative and manipulative investigations have provided at times equivocal or contrasting results [10]. The relationships between biodiversity and functioning of marine ecosystems are most often positive [11], so that any biodiversity loss could result in a decrease of the ecosystem functioning [12] and, consequently, in a lower provision of goods and services to the humans [2], [13]. A biodiversity loss can thus potentially impair the ecosystems’ capacity to sustain humanity [14]. This is of particular concern in tropical and subtropical environments which host an important fraction of the biodiversity of the coastal oceans and are among the world regions that will experience the earliest emergence of historically unprecedented climates and changes in biodiversity [15].

Either correlative or manipulative approaches for assessing the shape and strength of the relationships between biodiversity and ecosystem functioning in marine ecosystems have been mostly carried out using the diversity of macro-fauna or macro-algae as the independent variable [16]. More recently, meiofauna, the most abundant benthic group of metazoans in marine ecosystems [17], have been utilized for investigating, though only with a correlative approach, the linkages between biodiversity and ecosystem functioning in deep-sea sediments [18], [19]. Among metazoan meiofauna, nematodes respond rapidly to many different sources of natural and anthropogenic disturbance affecting sedimentary environments; thus, also due to their high abundance, species and functional (trophic) diversity, nematodes are an ideal tool to investigate the relationships between biodiversity and ecosystem functioning [20]. Meiofauna play also a key ecological role in linking detrital (and prokaryotic) resources with higher trophic levels: in fact most of the meiofaunal taxa eat microalgae, prokaryotes and detritus and, at the same time, it is known that meiofauna are a food source for macrofauna and fishes [21]–[25]. Meiofauna and nematodes, based on laboratory and *in situ* experiments, are in fact able to influence microbial activities and to graze their production [17], [26]. Meiofauna and nematodes are also very sensitive to the broad variations in natural environmental conditions (tidal influence, river inputs and local rainfall, food availability, sediment chemistry, bottom current regimes, habitat heterogeneity, among the others) [17], [27] that characterize marine sediments across all spatial scales and water depths [28]–[31].

The functioning of marine sedimentary ecosystems relies on the rates of organic matter cycling, which is also related to the production of heterotrophic prokaryotes: in turn, these are related to the food quantity and food availability [32], [33]. This holds true, in particular for highly detrital ecosystems like seagrass beds [34], mangroves [35] and coral reefs [36], where organic detritus, through the so-called microbial loop, is firstly incorporated into prokaryotic biomass, then enters higher trophic levels through bacterivorous, detritivorous and deposit feeders inhabiting the benthos [32]. Thus, it can be hypothesized that, in those ecosystems, the shape and strength of the relationship between biodiversity and ecosystem function could be influenced by the quantity and bioavailability of food resources.

To provide insights about the relationships between meiofaunal biodiversity and the ecosystem functioning in different tropical sedimentary habitats, we analyzed the richness of meiofaunal taxa and of nematode species (biodiversity) along with sedimentary organic matter quantity and nutritional quality, and prokaryotic heterotrophic production (functioning) across three habitats (i.e., seagrass, mangrove and reef sediments) in the Caribbean, Red and Celebes Seas. We also explored how some of the potential drivers of biodiversity considered in our study (longitude, quantity and quality of trophic sources) could influence the biodiversity-ecosystem functioning linkage. By comparing different habitats and regions we posed the following questions: i) how the different target variables vary among habitats and regions?; ii) whether and how changes the relationship between biodiversity and ecosystem functioning across different habitats?.

## Materials and Methods

### Ethical Statement

No special permits were requested at the time of sampling as in all regions sediment samples were collected out of protected areas and respecting local legislation. No protected species or taxa were sampled.

### Study Areas and Sampling Activities

**Figure 1:**
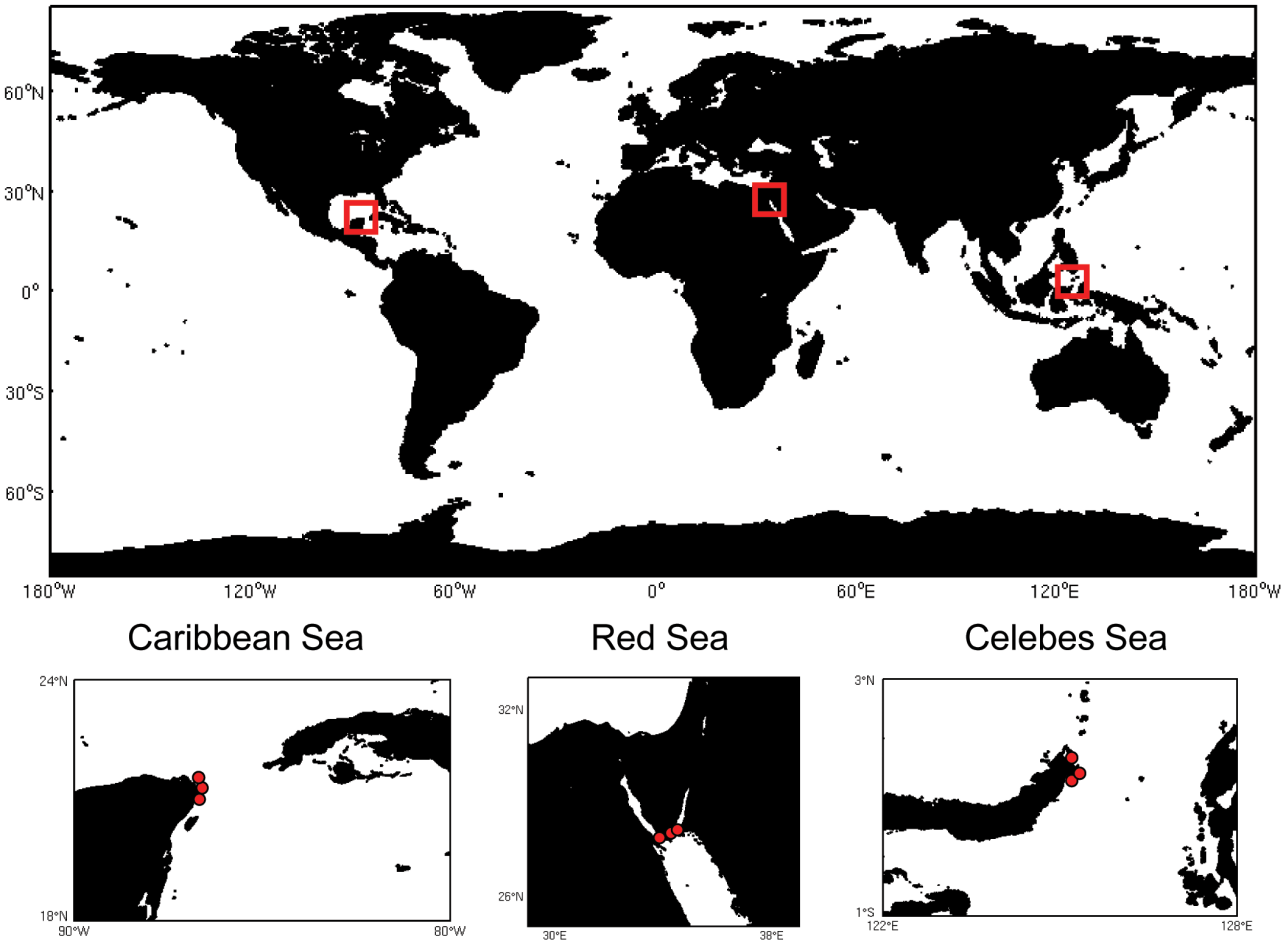
Sampling areas and location of the investigated regions with details of the positioning of each habitat type.

The sampling strategy included a total of nine sampling sites, from three tropical regions: the Caribbean, Red and Celebes Seas (Fig. 1, Table 1). In each region, three habitat types were visited once each: seagrass, mangrove and coral reef sediments.

**Table 1:** Biochemical composition of sedimentary organic matter, prokaryotic biomass and C production in the investigated habitats and regions.

Region	Habitat	Latitude	Longitude	Depth	Chlorophyll-*a*	Phaeopigment	Protein	Carbohydrate	Lipid	Biopolymeric Carbon	Prokaryotic biomass	Prokaryotic heterotrophic production
		° N	°E	m	µg g^−1^	µg g^−1^	mg g^−1^	mg g^−1^	mg g^−1^	mgC g^−1^	µgC g^−1^	µgC g^−1^ d^−1^
Caribbean	Seagrass	21.6	−87.0	0.50	1.4±0.2	8.7±1.5	1.44±0.37	1.66±0.42	0.54±0.11	1.77±0.42	1.73±0.42	1.73±0.42
	Mangrove	21.3	−86.8	0.50	4.8±0.1	92.6±5.0	10.46±1.42	21.24±3.29	8.36±1.00	19.89±0.89	71.4±11.1	1.78±0.28
	Reef	20.5	−86.8	3.00	2.7±2.8	34.7±5.9	0.83±0.14	2.49±0.22	0.52±0.01	1.79±0.15	0.47±0.04	0.21±0.02
Red	Seagrass	28.2	34.4	0.50	0.2±0.1	16.4±4.7	0.61±0.15	0.53±0.03	0.03±0.00	0.54±0.06	1.04±0.34	1.04±0.34
	Mangrove	28.2	34.4	0.50	0.1±0.0	13.4±1.4	0.28±0.06	0.07±0.04	0.02±0.00	0.18±0.02	1.71±0.24	1.20±0.17
	Reef	28.2	34.4	2.00	0.4±0.1	49.9±8.0	0.67±0.09	0.10±0.04	0.05±0.03	0.41±0.07	0.36±0.08	0.16±0.03
Celebes	Seagrass	1.6	125.4	0.50	2.2±1.0	20.4±8.9	2.72±0.46	7.23±0.61	1.05±0.26	7.17±0.45	4.04±2.04	4.04±2.04
	Mangrove	1.5	125.2	0.50	0.7±0.3	4.0±1.9	1.78±0.21	1.63±0.22	0.63±0.31	2.35±0.19	6.38±0.53	2.23±0.18
	Reef	1.6	125.5	5.00	1.0±0.3	5.8±1.1	0.44±0.12	0.80±0.10	0.20±0.04	0.90±0.08	8.24±0.59	3.59±0.26

In the Caribbean Sea, sediment samples were collected in the east coast of the Yucatan peninsula (Mexico) from a seagrass bed (Isla Mujeres), a coral reef (Xelha) and a mangrove forest (dominated by *Rizophora sp*.). In the Red Sea, sediment samples were collected in the Nabq lagoon from a seagrass bed, a mangrove forest dominated by *Avicennia marina* and a coral reef. In the Celebes Sea (Sulawesi Island, Indonesia), sediment samples were collected from a seagrass bed (dominated by *Enhalus acoroides*), a mangrove forest and a coral reef.

Sampling sites (each representing one type of habitat) within a region were 10–35 kilometers distance apart. At all sampling sites, replicate sediments within each habitat have been collected from three 0.5×0.5 m quadrates randomly selected from one 5×5 m area per habitat. The choice of this sampling area was constrained by the limited capability of operational movement in the three regions. Although this could have possibly reduced the ability to identify precisely the patterns of variability at the habitat scale, this design allowed the collection of sediments from the different habitats in each region within a reasonable time interval (hours-days).

Sediment samples were collected by scuba diving using Plexiglas manual corers (internal diameter 4.6 cm). The top first centimeter of three independent corers was collected for the determination of the quantity and biochemical composition of sedimentary organic matter, prokaryotic abundance, biomass and C production.

All samples for organic matter and biomass determinations were stored at −20°C until the analyses in the laboratory, whereas samples for the determination of prokaryotic heterotrophic production were immediately incubated as described below.

Three additional independent corers, collected for the analyses of meiofaunal abundance, biomass and biodiversity, were preserved in buffered formalin (4%), stained with Rose Bengal (0.5 gL^−1^) and stored at 4°C until analysis.

### Sedimentary Organic Matter

The sedimentary contents of phytopigments and biopolymeric C pools reflect the overall trophic conditions of marine coastal sediments, whereas the algal fraction of biopolymeric C pools reflects the food quality of sedimentary detritus [37]. Chloroplastic pigments (chlorophyll-*a* and phaeopigments) were analyzed fluorometrically [38]. Pigments were extracted from the top centimeter of each core with 90% acetone (24 h in the dark at 4°C). After centrifugation (800×g), the supernatant was used to determine the functional chlorophyll-*a* and acidified with 0.1 N HCl to estimate the amount of phaeopigments. Chlorophyll-*a* and phaeopigment concentrations were summed up and reported as total phytopigment concentrations. Total phytopigments, after transformation into C equivalents using 30 as a conversion factor [39], are thus used as an estimate of the organic material of algal origin including either the living (chlorophyll-*a*) and senescent/detrital (phaeopigment) fractions [40].

Protein, carbohydrate and lipid analyses were carried out on the top 1 cm of three independent corers from each site using photometric protocols [39]. Protein, carbohydrate and lipid sedimentary contents were converted to carbon equivalents by using the following conversion factors: 0.49, 0.40 and 0.75 mg C mg^−1^, respectively and their sum referred as biopolymeric C (BPC) [37].

### Prokaryotic Biomass and Heterotrophic Production

Prokaryotic biomass was determined from the prokaryotic cell biovolume and abundance by epifluorescence microscopy. To estimate the cell biovolume we examined at least 100 cells for sediment sample classifying prokaryotes into three different size classes: small (<0.065 µm^3^), medium (0.065–0.320 µm^3^) and large (0.320–0.574 µm^3^) and the biovolume was converted into carbon content assuming 310 fg C µm^3^ as a conversion factor [41]. The total prokaryotic abundance was determined using epifluorescence microscopy [39]. Briefly, sediment subsamples (0.25 g) were added with 1125 µl of 2% formalin and 125 µl of pyrophosphate (final concentration, 5 mM). Then, these were treated with ultrasounds (three times for 1 min; Branson sonifier 2200, 60 W) to increase the extraction efficiency and diluted 250–500 times using autoclaved and 0.2 ml pre-filtered seawater. One ml of the supernatant was stained with Acridine Orange (final concentration, 0.01%), for 5 min in the dark) and filtered under vacuum (<100 mmHg) using 0.2 µm pore size black Nuclepore Polycarbonate filters. The filters were then washed twice with 3 ml of 0.2 µm pre-filtered and sterilized reagent grade water, mounted on microscope slides, and analyzed under an epifluorescence microscope (Zeiss Axioskop 2; magnification, ×1000). For each slide, at least 10 fields were observed, for a total of at least 400 cells counted per filter. Prokaryotic abundance is expressed as cells g^−1^ dry sediment (after desiccation at 60°C for 24 h).

Benthic prokaryotic heterotrophic production was measured by ^3^H-leucine incorporation [42]. Sediment sub-samples (200 µl), added to a saturating aqueous solution of ^3^H-leucine (6-µCi final concentration per sample), were incubated for 1 h in the dark at in situ temperature. After incubation, prokaryotic C incorporation was stopped with 1.7 ml of 80% ethanol before scintillation counting. Sediment blanks were made adding ethanol immediately before ^3^H-leucine addition. Data were normalized to sediment dry weight after desiccation (60°, 24 h).

### Meiofaunal Abundance and Biomass

For metazoan meiofaunal extraction, sediment samples were sieved through a 500 µm and a 30 µm mesh, respectively, to retain the smallest organisms. The fraction remaining on the latter sieve (including organisms with a size of 30–500 µm) was resuspended and centrifuged three times with Ludox HS 40 (diluted with tap water to arrange density to 1.18 g cm^−3^) for muddy samples, whereas for sandy samples, meiofauna were extracted using decantation (repeated 10 times for 95% of efficiency) [39], [43]. After the extraction the sediments have been carefully checked to search for remnant organisms. No organisms were observed in the residual sediments after the treatment with Ludox or decantation. All metazoan animals were counted and classified per taxon under a stereomicroscope using Delfuss cuvettes, after staining with Rose Bengal (0.5 g L^−1^). For the determination of meiofaunal biomass, we calculated the individual biomass of all animals belonging to different taxa. Nematode biomass was calculated from biovolume (n = 100 per sample) using Andrassy’s formula (V = L × W^2^ × 0.063 × 10^−5^; body length L in µm and width W in µm). For all other taxa, the biovolume was measured for all of the specimens encountered. Body volume was derived from measurements of body length (L; in mm) and width (W; in mm) using the formula V = L × W^2^ × C; where C is the approximate conversion factor for each meiofaunal taxon [44]. The body volume was multiplied by an average density (1.13 g cm^−3^) to obtain the biomass (µg DW) assuming that the dry : wet weight ratio was 20–25%, and the C content was considered as 40% of the dry weight [44].

### Nematode Biodiversity

Nematode species richness and assemblage composition was determined only in the Celebes and Caribbean Seas. For the analysis of nematode diversity, 100 specimens (or all of the retrieved nematodes if <100) were randomly picked up from each of the three independent replicates at each sampling station. Nematodes were mounted on slides (following the formalin-ethanol-glycerol technique to prevent dehydration) [39] and identified to species level. Species identity was not considered in this study but, for the purposes of estimating species richness and other diversity indexes, the different morphotypes belonging to each genus were indicated as sp1, sp2, sp3 and considered as separate species [45]. Nematode species richness was estimated as the total number of species identified in each habitat. Since species richness is strongly influenced by the number of the individuals identified, to standardize the values of nematode diversity, the species-abundance data were converted into rarefaction diversity indices [46], [47]. The expected number of species for a theoretical sample of 51 nematode specimens, ES(51), was calculated. Previous studies have shown that this approach enables the provision of robust data on species richness. Although it is far from being perfect [48], the expected species number is the density-independent index most commonly used for the comparison of areas with a non-standardized sample size [31], [49]. The Pielou’s index (J’) was also calculated [50].

We measured also the beta diversity of meiofaunal and nematode assemblages among habitats within each region and among regions within each habitat using the SIMPER analysis based on Bray-Curtis matrixes, and expressed as percentage of dissimilarity [48]. Taxon and species-abundance data were presence/absence transformed prior to the analysis to search for specific assemblages in each habitat and region, irrespectively of the taxon and species relative abundance. Diversity indexes and the dissimilarity estimates were calculated using the routine DIVERSE and SIMPER, respectively included in the PRIMER6+ software [51].

Functional diversity of nematodes was estimated using the Index of Trophic Diversity (ITD) calculated as ITD = g_1_^2^+g_2_^2^+g_3_^2^…+g_n_^2^, where *g* is the relative contribution of each trophic group to the total number of individuals and *n* is the number of trophic groups (with n = 1–4) [52]. Nematodes were divided into four groups as follows: (1A) no buccal cavity or a fine tubular one -selective (bacterial) feeders; (1B) large but unarmed buccal cavity-non-selective deposit feeders; (2A) buccal-cavity with scraping tooth or teeth-epistrate or epigrowth (diatom) feeders; (2B) buccal cavity with large jaws-predators/omnivores [52]. For four trophic guilds, the Index of Trophic Diversity ranges from 0.25 (highest trophic diversity; i.e., the four trophic guilds account for 25% each) to 1.0 (lowest diversity; i.e., one trophic guild accounts for 100% of nematode abundance) [43].

### Ecosystem Functioning

Resembling one of the approach used for assessing biodiversity and ecosystem functioning relationships in the deep sea [18], we used prokaryotic heterotrophic production as proxy of the benthic ecosystem functioning and related it to biodiversity, estimated in terms of richness of meiofaunal taxa and nematode expected species number [ES(51)].

### Statistical Analyses

To assess separately the differences in the quantity of sedimentary trophic resources, prokaryotic variables (biomass and heterotrophic production), total meiofaunal abundance, biomass, and richness of taxa among habitats within each region, and among regions within each habitat, we used two-way permutational analyses of variance (PERMANOVA) under a reduced model. The analyses were carried out using Habitat (3 fixed levels: mangrove, reef and seagrass; orthogonal to Region) and Region (3 fixed levels: Celebes, Red and Caribbean Seas) as main sources of variance. The same design was used for the analysis of nematode species diversity, but, in this case, the factor region included two levels only (Celebes and Caribbean Seas).

The same design has been used as the basis for a distance-based permutational multivariate analyses of variance (PERMANOVA) [53] to test variations among habitats and regions in the biochemical composition of sedimentary organic matter (based on Euclidean distance matrices), in the meiofaunal and the nematode assemblages (based on Bray Curtis similarity matrices after presence/absence transformations). The analyses were carried out using the permutation of residuals under a reduced model. In both uni- and multivariate tests, significant terms were investigated using a posteriori pair-wise comparisons with the PERMANOVA *t* statistic and 999 permutations.

The variations in meiofaunal communities and nematode assemblages among regions and habitats were illustrated using bi-plots produced after a canonical analysis of principle coordinates (CAP).

Biodiversity vs. ecosystem functioning relationships were assessed using distance-based analyses for a linear model using the routine DISTLM before and after the removal of covariates associated with the geographical location (longitude) and the availability of food resources (here evaluated in terms of biopolymeric C and protein to carbohydrate ratio), as synthetic descriptors of quantity and nutritional quality of food, respectively.

PERMANOVA, CAP and DISTLM tests were carried out using the homonymous routines included in the PRIMER6+ software [51].

## Results

### Sedimentary Organic Matter

The results of the two-way PERMANOVA carried out including the three habitat types in the Caribbean, Red and Celebes Seas revealed the presence of a significant effect of the interaction Habitat × Region for phytopigment, biopolymeric C and prokaryotic biomass (Table 2).

**Table 2:** Results of PERMANOVA and pairwise test for differences in sedimentary organic matter quantity and biochemical composition, prokaryotic biomass and heterotrophic production.

						Pairwise tests	
	Source	DF	MS	Pseudo-F	P	Region (Habitat)	Habitat (Region)
**Phytopigments**	**Region**	2	3.8	57.14	***	Car [M>R>S]	S [Car = Red = Cel]
	**Habitat**	2	0.4	6.07	*	Red [R>S = M]	M [Car>Red>Cel]
	**Region × Habitat**	4	3.1	46.71	***	Cel [S>M = R]	R [Red = Car>Cel]
	**Residuals**	18	0.1				
**Proteins**	**Region**	2	1.8	187.69	***	Cel [S>M>R]	S [Cel>Car>Red]
	**Habitat**	2	1.2	127.98	***	Car [M>S>R]	M [Car>Cel>Red]
	**Region × Habitat**	4	1.2	127.14	***	Red [R = S>M]	R [Car>Cel ]
	**Residuals**	18	0.0				
**Carbohydrates**	**Region**	2	5.8	736.42	***	Car [M>R = S]	S [Cel>Car>Red],
	**Habitat**	2	1.3	163.86	***	Red [S>R = M]	M [Car>Cel>Red]
	**Region × Habitat**	4	2.4	301.53	***	Cel [S>M>R]	R [Car>Red>Cel]
	**Residuals**	18	0.0				
**Lipids**	**Region**	2	2.2	278.69	***	Car [M>S = R]	S [Cel>Car>Red]
	**Habitat**	2	1.2	145.26	***	Red [R>S>M]	M [Car>Cel>Red]
	**Region × Habitat**	4	1.2	144.26	***	Cel [S>M = R]	R [Car>Cel>Red]
	**Residuals**	18	0.0				
**Biopolymeric C**	**Region**	2	4.6	1076	***	Car [M>R = S]	S [Cel>Car>Red]
	**Habitat**	2	1.5	346.56	***	Red [R = S>M]	M [Car>Cel>Red]
	**Region × Habitat**	4	2.1	501.85	***	Cel [S>M>R]	R [Car>Cel>Red]
	**Residuals**	18	0.0				
**Biochemical composition**	**Region**	2	20.2	42.83	***	Car [M≠R = S]	S [Cel≠Car≠Red]
	**Habitat**	2	10.1	21.43	***	Red [R≠[M = S]	M [Cel≠Car≠Red]
	**Region × Habitat**	4	15.2	32.14	***	Cel [M≠S≠R]	R [Cel≠Red]
	**Residuals**	18	0.5				
**Prokaryotic biomass**	**Region**	2	9.2	138.3	***	Car [S = M>R]	S [Cel>Car = Red]
	**Habitat**	2	11	163.5	***	Red [S = M>R]	M [Car = Cel>Red]
	**Region × Habitat**	4	5.9	88.7	***	Cel [R>M = S]	R [Cel>Car = Red]
	**Residuals**	18	0				
**Prokaryotic heterotrophic production**	**Region**	2	2.0	64.7	***	Car [S = M>R]	S [Cel> Red = Car]
	**Habitat**	2	0.5	18.7	***	Red [S = M>R]	M [Car = Cel>Red]
	**Region × Habitat**	4	0.3	10.6	***	Cel [S = R>M]	R [Cel>Car = Red]
	**Residuals**	18	0.0				

DF = degrees of freedom; MS = mean squares; Pseudo-F = F statistic; P = probability level; *** = P<0.001; * = P<0.05; ns: not significant. Car = Caribbean Sea; Red = Red Sea; Cel = Celebes Sea; M = mangrove; R = reef; S = seagrass.

**Figure 2:**
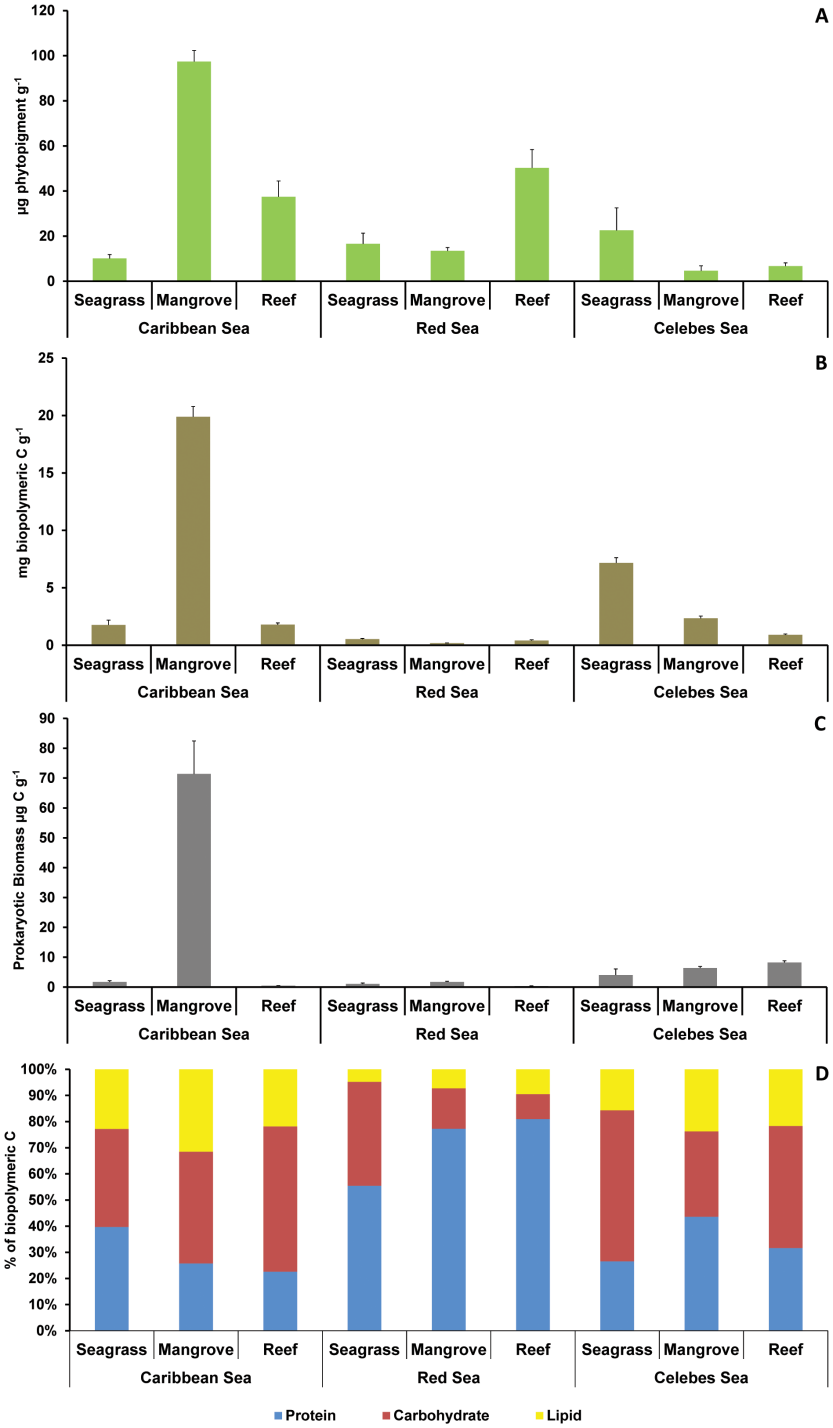
Biochemical composition of sedimentary organic matter. Reported are the concentrations of A) total phytopigments, B) biopolymeric carbon, C) prokaryotic biomass, and D) the biochemical composition (as percentage contribution of the biopolymeric carbon content) of the sedimentary organic matter in seagrass, mangrove and reef habitats of the Caribbean, Red and Celebes Seas. Reported are mean values ± standard deviation.

In the Caribbean Sea, the highest phytopigment and biopolymeric C contents occurred in mangrove sediments; in the Red Sea the highest total phytopigment contents occurred in reef sediments, whereas in the Celebes Sea the highest contents were observed in the seagrass sediments (Fig. 2A–C). Comparing seagrass habitats in different regions, phytopigment and biopolymeric C contents in the Celebes Sea were significantly higher than those in both the Caribbean and the Red Seas, whereas values in the Caribbean mangrove sediments were consistently higher than those in the Celebes and Red Seas.

Prokaryotic biomass in seagrass sediments did not vary significantly across regions (Table 2). In mangrove sediments prokaryotic biomass showed the highest values in the Caribbean Sea and the lowest in the Red Sea, whereas in reef sediments it was significantly higher in the Celebes Sea than in the Caribbean and Red Seas (Table 2).

The pairwise tests revealed also the presence of significant differences in the biochemical composition of sediments among regions (for mangrove and seagrass sediments) (Table 2). The reef sediments of the Celebes and Caribbean Seas exhibited a similar biochemical composition characterized by the dominance of carbohydrates, whereas the protein fraction dominated in the Red Sea reef sediments (Fig. 2D).

### Prokaryotic Biomass and Heterotrophic Production

**Figure 3:**
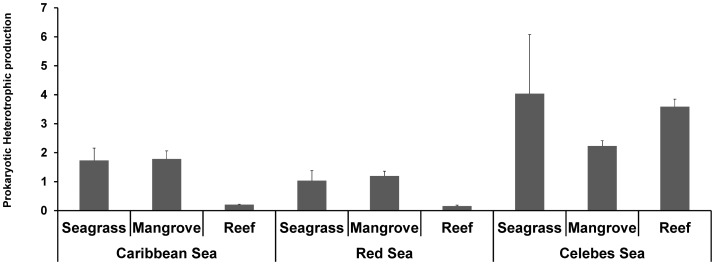
Ecosystem functioning as prokaryotic heterotrophic production (µgC g ^−**1**^ **d** ^−**1**^ **) in seagrass, mangrove and reef habitats of the Caribbean, Red and Celebes Seas.**

Prokaryotic biomass and heterotrophic production values were characterized by a significant effect of the Habitat × Region interaction (Table 2). In the Caribbean and Red Seas, prokaryotic heterotrophic production values in seagrass and mangrove sediments were significantly higher than those in reef sediments, whereas in the Celebes Sea the mangrove sediments were characterized by values significantly lower than those in seagrass and reef sediments (Fig. 3). Overall, prokaryotic heterotrophic production in the sediments of the Celebes Sea were generally and significantly higher than those in the two other regions.

### Meiofaunal Abundance, Biomass and Community Composition

Data on meiofaunal abundance, and richness of taxa, nematode diversity as species richness, ES(51), evenness (Pielou’s J) and index of trophic diversity in all habitats and regions are reported in Table 3.

**Table 3:** Meiofaunal abundance and richness of taxa, nematode diversity as Species Richness, ES(51), evenness (Pielou’s J) and index of trophic diversity in all habitats and regions.

Region	Habitat	Latitude	Longitude	Depth	Meiofaunal abundance	Richness ofmeiofaunal taxa	SpeciesRichness	Expected species number	Pielou’s evenness	Trophic diversity
		° N	°E	m	Ind. 10 cm^−2^			ES(51)	J	ITD
Caribbean	Seagrass	21.6	−87.0	0.50	1268±731	11.3±4.2	33	14.1±4.3	0.82±0.05	0.49±0.04
	Mangrove	21.3	−86.8	0.50	2474±117	5.0±1.0	13	6.7±0.1	0.48±0.01	0.80±0.02
	Reef	20.5	−86.8	3.00	2871±1307	9.0±1.0	28	10.9±2.0	0.68±0.00	0.38±0.02
Red	Seagrass	28.2	34.4	0.50	1475±166	11.0±1.7	n/a	n/a	n/a	n/a
	Mangrove	28.2	34.4	0.50	343±41	4.0±1.0	n/a	n/a	n/a	n/a
	Reef	28.2	34.4	2.00	488±253	6.0±2.0	n/a	n/a	n/a	n/a
Celebes	Seagrass	1.6	125.4	0.50	1604±267	16.3±2.5	59	24.5±4.0	0.90±0.03	0.44±0.08
	Mangrove	1.5	125.2	0.50	627±192	9.7±1.5	40	16.1±2.9	0.79±0.04	0.43±0.08
	Reef	1.6	125.5	5.00	706±271	17.0±1.0	77	27.5±1.2	0.90±0.05	0.45±0.05

n/a = not analyzed.

**Figure 4:**
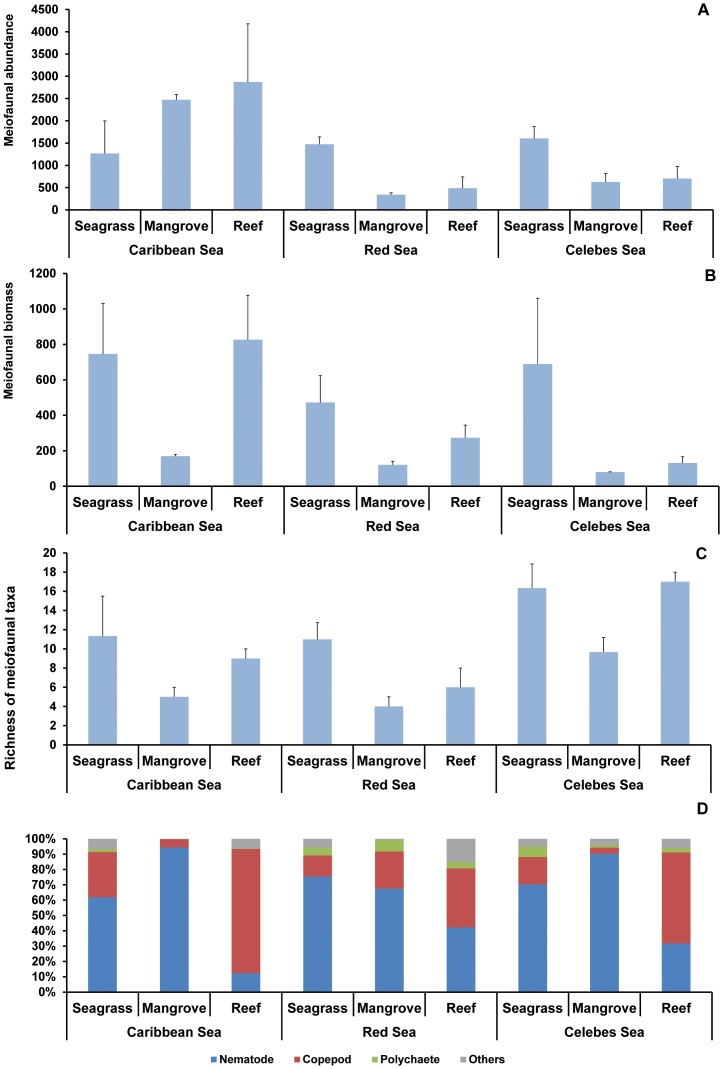
Meiofaunal assemblages. Illustrated are meiofaunal: A) abundance (ind 10 cm^−2^), B) biomass (µgC 10 cm^−2^), C) richness of taxa, and D) assemblage composition in seagrass, mangrove and reef habitats of the Caribbean, Red and Celebes Seas. Reported are average values ± standard deviation.

The results of the PERMANOVA on meiofaunal abundance revealed a significant effect of the interaction Habitat × Region (Table 4, Fig. 4A). In more detail, the pairwise tests revealed that, in both the Celebes and Red Seas, meiofaunal abundance was significantly higher in seagrass than in mangrove and reef sediments, whereas in the Caribbean Sea values did not vary significantly among habitats. The pairwise tests revealed also that the meiofaunal abundance in both mangrove and reef habitats was significantly higher in the Caribbean than in the Celebes and Red Seas. Meiofaunal abundance in seagrass sediments did not vary significantly across regions.

**Table 4:** Results of PERMANOVA testing for differences in meiofaunal abundance, biomass, richness of taxa and assemblages composition among habitats across regions and among regions across habitats.

						Pairwise tests	
	Source	DF	MS	Pseudo-F	P	Region (Habitat)	Habitat (Region)
**Meiofaunal abundance**	**Habitat**	2	0.74	5.54	**	Car [S = M = R]	S [Car = Red = Cel]
	**Region**	2	3.31	24.9	***	Red [S>M = R]	M [Car>Cel>Red]
	**Habitat × Region**	4	1.3	9.8	***	Cel [S>M = R]	R [Car>Cel = Red]
	**Residuals**	18	0.13				
**Meiofaunal Biomass**	**Habitat**	2	2.0	6.91	***	Car [R = S>M]	S [Car = Red = Cel]
	**Region**	2	5.0	17.77	***	Red [S = R>M]	M [Car>Red = Cel]
	**Habitat × Region**	4	0.6	2	ns	Cel [S>R = M]	R [Car>Red = Cel]
	**Residuals**	18	0.3				
**Richness of meiofaunal taxa**	**Habitat**	2	135.81	33.04	***	Car [R = S>M]	S [Cel>Red = Car]
	**Region**	2	103.70	25.23	***	Red [R = S>M]	M [Cel>Car = Red]
	**Habitat × Region**	4	7.70	1.87	ns	Cel [R = S>M]	R [Cel>Car = Red]
	**Residuals**	18	4.11				
**Assemblages composition**	**Habitat**	2	3430	5.67	***	Car [S = M = R]	na
	**Region**	2	2759	2.80	ns	Red [S≠M = R]	na
	**Habitat × Region**	4	985	1.63	ns	Cel [M≠S = R]	na
	**Residuals**	18	605				

Results of the pairwise tests among levels of the relevant factors are also reported. DF = degrees of freedom; MS = mean squares; Pseudo-F = F statistic; P = probability level; *** = P<0.001; ** = P<0.01; ns: not significant. Car = Caribbean Sea; Red = Red Sea; Cel = Celebes Sea; M = mangrove; R = reef; S = seagrass; na = not applicable.

Meiofaunal biomass varied significantly among habitats (within the same region) and among regions (comparing the same habitat) (Table 4, Fig. 4B). Meiofaunal biomass was generally lower in mangrove sediments (range 79–170 µgC 10 cm^−2^) than in the other two habitats (range 131–826 µgC 10 cm^−2^). Significantly higher values of meiofaunal biomass were observed in the Caribbean followed by the Celebes and the Red Seas (Table 4).

The richness of meiofaunal taxa (Fig. 4C) ranged from 4 (in the mangrove sediments of the Red Sea) to 17 (in the reef sediments of the Celebes Sea) and varied significantly among habitats and among regions (Table 4). In more detail, the pairwise tests revealed that in all regions the richness of meiofaunal taxa in seagrass and reef sediments was consistently and significantly higher than in mangrove sediments. The richness of meiofaunal taxa in the Celebes Sea was significantly higher than that in the Caribbean and Red Seas (Table 4, Fig. 4C).

In all habitats and regions, with exception of reef sediments of the Celebes and Caribbean Seas, nematodes were the dominant taxon (52–97%), followed by copepods (3–25%) and polychaetes (0–13%) (Fig. 4D). Only reef sediments of the Caribbean and Celebes Seas were dominated by copepods (78 and 54% of total meiofaunal abundance, respectively). The contribution of all other identified taxa (acarians, amphipods, bivalves, cladocerans, cnidarians, cumaceans, gastrotrichs, gastropods, isopods, kinorinchs, decapods larvae, oligochaetes, ostracods, priapulians, tanaidaceans, tardigrades, termosbanaceans and turbellarians) varied from 0 to 25% of the total meiofaunal abundance (Fig. S1).

**Figure 5:**
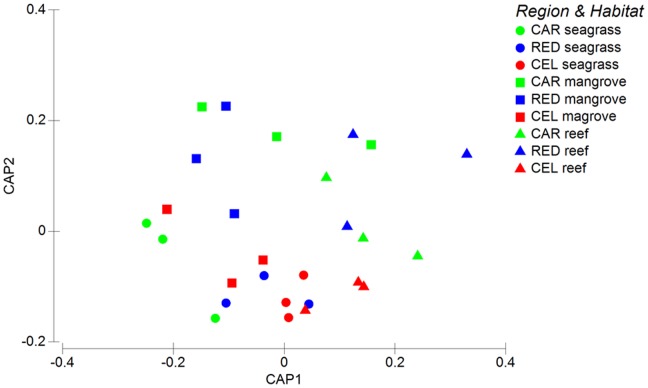
Bi-plots after the canonical analysis of principal coordinates illustrating differences in the composition of total meiofaunal taxa in the sediments of the investigated oceanic regions and habitats. CAR = Caribbean Sea (green); CEL = Celebes Sea (red); RED = Red Sea (blue).

The PERMANOVA test showed that the composition of meiofaunal communities varied significantly only among habitats (Table 4). Moreover, the pairwise tests revealed that differences in the composition of meiofaunal communities were not consistent among habitats within each region (Table 4). Consequently, the results of the CAP analyses revealed that the differences among regions in the composition of meiofaunal assemblages were not well defined (Fig. 5).

In the Caribbean Sea, the highest meiofaunal beta diversity occurred between seagrass and mangrove sediments (60%). In the Red Sea, the highest meiofaunal beta diversity occurred between seagrass and reef sediments (44%), whereas in the Celebes Seas the highest beta diversity occurred between mangrove and reef sediments (29%) (Table S1). When contrasting the regions, the highest meiofaunal beta diversity among seagrass sediments is observed between the Red and the Celebes Sea (30%), whereas for mangrove sediments the highest beta diversity is observed between the Caribbean and Celebes Sea (61%). For reef sediments the highest meiofaunal beta diversity is observed between the Red and Celebes Seas (48%).

### Nematode Biodiversity

**Figure 6:**
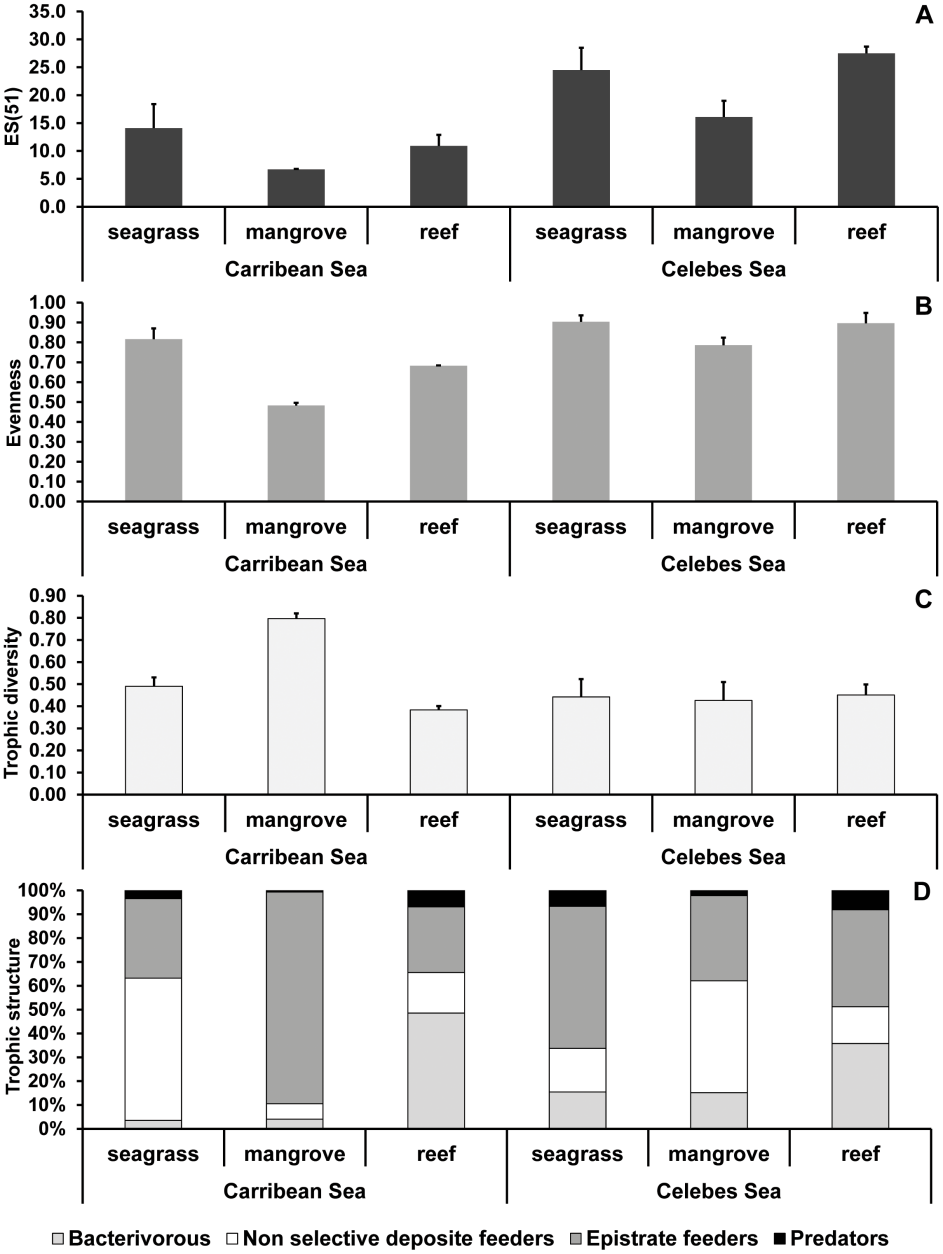
Nematode diversity. Illustrated are A) expected species number [ES(51)], B) evenness (Pielou’s J), C) index of trophic diversity (ITD) and D) trophic composition of nematode assemblages in seagrass, mangrove and reef habitats of the Caribbean, Red and Celebes Seas.

The results of PERMANOVA conducted on nematode species richness and expected species number [ES(51)] revealed significant effects of the factors Region and Habitat, but no significant interaction effects (Table 5). Overall, in both the Celebes and Caribbean Seas the mangrove sediments displayed consistently and significantly lower values of nematode species richness, expected species number and evenness values (Table 5; Fig. 6). In all habitats, nematode expected species number and evenness were consistently higher in the Celebes than in the Caribbean Sea (with the only exception of evenness in seagrass sediments) (Table 5; Fig. 6A–B). Values of the index of trophic diversity did not vary among habitats in the Celebes Sea, whereas decreased from mangrove to seagrass and reef sediments in the Caribbean Sea. Values of the trophic diversity index varied significantly among regions only in mangrove sediments (Fig. 6C). In seagrass and reef sediments of the Caribbean Sea the selective (bacterial) and non-selective deposit feeders cumulatively represented more than 60% of the total nematode abundance, whereas in mangrove sediments dominated the epistrate feeders (more than 80% of the total abundance, Fig. 6D). In mangrove and reef sediments of the Celebes Sea the selective and non-selective deposit feeders cumulatively represented more than 50% of the total nematode abundance, whereas in seagrass sediments dominated the epistrate feeders (more than 60% of the total abundance). In both the Celebes and Caribbean Seas and in all habitats predator nematodes represented a minor fraction of the total nematode abundance (range 1–7%).

**Table 5:** Results of PERMANOVA testing for differences in nematode biodiversity, as nematode Species Richness, ES(51), Pielou’s evenness and index of trophic diversity (ITD), and nematode assemblage composition among habitats across regions and among regions across habitats.

						Pairwise test	
	Source	DF	MS	Pseudo-F	P	Region (Habitat)	Habitat (Region)
**Species richness**	**Region**	1	2.39	59.13	**	Car [S = R>M]	S [Cel>Car]
	**Habitat**	2	0.3	7.45	*	Cel [S = R>M]	M [Cel>Car]
	**Region × Habitat**	2	0.03	0.79	ns		R [Cel>Car]
	**Residuals**	12	0.04				
**ES(51)**	**Region**	1	2.44	93.8	***	Car [R = S>M]	S [Cel>Car]
	**Habitat**	2	0.5	19.36	***	Cel [R = S>M]	M [Cel>Car]
	**Region × Habitat**	2	0.05	1.79	ns		R [Cel>Car]
	**Residuals**	12	0.03				
**Evenness**	**Region**	1	0.06	148.78	***	Car [S>R>M]	S [Car = Cel]
	**Habitat**	2	0.03	67.18	***	Cel [R = S>M]	M [Cel>Car]
	**Region × Habitat**	2	0.01	17.29	***		R [Cel>Car]
	**Residuals**	12	0.00				
**ITD**	**Region**	1	0.02	16.37	***	Car [M>S>R]	S [Cel = Car]
	**Habitat**	2	0.02	16.27	***	Cel [M = R = S]	M [Car>Cel]
	**Region × Habitat**	2	0.03	21.33	***		R [Cel = Car]
	**Residuals**	12	0.00				
**Nematode assemblage**	**Region**	1	7074	4.62	**	Car [S≠M≠R]	S [Cel = Car]
	**Habitat**	2	7267	1.43	ns	Cel [S = M = R]	M [Cel≠Car]
	**Region × Habitat**	2	5071	3.31	***		R [Cel = Car]
	**Residuals**	12	1532				

Results of the pairwise tests among levels of the relevant factors are also reported. df = degrees of freedom; MS = mean squares; Pseudo-F = F statistic; *** = P<0.001; ** = P<0.01; * = P<0.05; ns: not significant. Car = Caribbean Sea; Red = Red Sea; Cel = Celebes Sea; M = mangrove; R = reef; S = seagrass.

**Figure 7:**
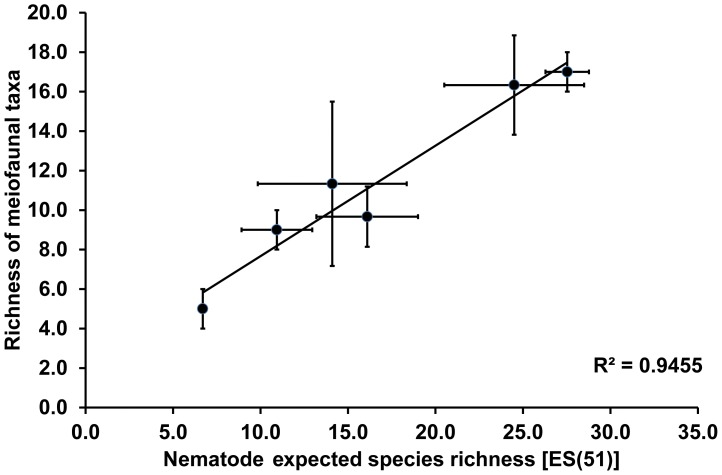
Relationship between richness of meiofaunal taxa and nematode biodiversity as ES(51) in tropical habitats from different oceanic regions. Illustrated are mean values ± standard deviation.

The richness of meiofaunal taxa was significantly and positively correlated with the number of nematode species as ES(51) (Fig. 7).

The results of PERMANOVA showed the presence of a significant effect of the interaction Habitat × Region on the composition of nematode assemblages (Table 5). The pairwise comparisons revealed that the composition of the nematode assemblages varied among regions only in mangrove sediments, whereas differences among habitats were consistently present only in the Caribbean Sea (Table 5). In the Celebes Sea, the nematode beta diversity among habitats was high and ranged from 64% (seagrass vs. reefs) to 79% (reefs vs. mangroves) (Table S1). In the Caribbean Sea the nematode beta diversity among habitats (74–93%) was slightly higher than that in the Celebes Sea (64–71%). Nematode beta diversity among regions was 68%, 77% and 83% in seagrass, mangrove and reef sediments, respectively. Overall the nematode beta diversity among the Caribbean and Celebes was 79%.

### Relationship between Biodiversity and Ecosystem Functioning

**Figure 8:**
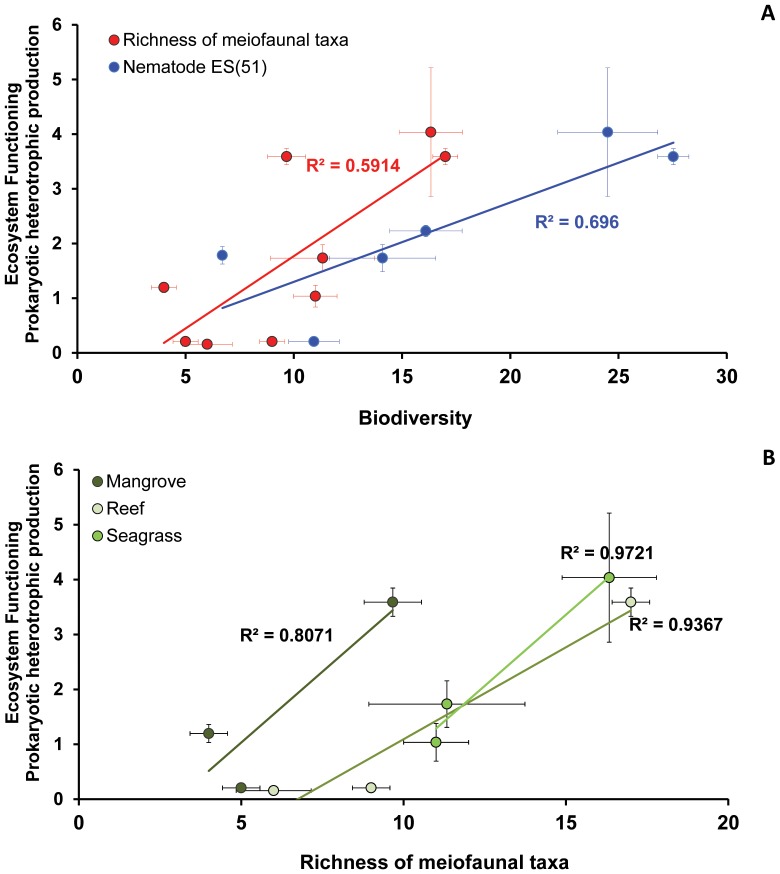
Relationship between biodiversity and ecosystem functioning. Illustrated are the relationships between: A) richness of meiofaunal taxa and prokaryotic heterotrophic production (µgC g^−1^ d^−1^); nematode diversity as ES(51) and prokaryotic heterotrophic production (µgC g^−1^ d^−1^); B) richness of meiofaunal taxa and prokaryotic heterotrophic production in different habitat types. Reported are R^2^ values. P<0.01 for all linear regressions. Error bars indicate standard deviations among replicates (n = 3).

The richness of meiofaunal taxa and the nematode expected species number [ES(51)] were significantly and positively related with prokaryotic heterotrophic production across all regions and in each habitat (Fig. 8A–B). The linear relationships between biodiversity and ecosystem functioning were not influenced by any of the tested covariates (i.e., longitude, biopolymeric C contents and the values of the protein to carbohydrate ratio) only when all data, irrespectively of region or habitat, were included in the analysis (Table 6).

**Table 6:** Effects of biodiversity, measured as richness of meiofaunal taxa and nematode expected species number ES(51), on ecosystem functioning, as prokaryotic heterotrophic production before and after the removal of the covariables’ effect: longitude, sediment biopolymeric organic C content and the protein to carbohydrate ratio.

Independent variable	R^2^	SS	Pseudo-F	P
**Richness of Meiofaunal Taxa**	0.56	39.25	32.39	***
**Covariates**	0.63	43.52	12.82	***
**Taxa Richness after removal of covariates**	0.72	6.53	7.37	*
**Nematode ES(51)**	0.73	39.97	42.24	***
**Covariates**	0.77	42.46	15.67	***
**Nematode ES(51) after the removal of covariates**	0.88	5.78	10.94	***

In the regression analyses, all tests were based on Euclidean distances calculated among observations from untransformed data, using all data from different regions and habitats. The following abbreviations are used: R^2^ = regression coefficient, SS = sum of squares, Pseudo-F = statistic F; P = probability level (*** = P<0.001; * = P<0.05).

## Discussion

### Meiofaunal and Nematode Biodiversity in Tropical Marine Sediments

Meiofaunal abundance and biomass values reported in this study fall within the range of those previously reported for the same regions (Caribbean Sea, Celebes Sea and Red Sea) or from other tropical and subtropical habitats [54]–[60]. Nonetheless, although our survey is limited to one replicate habitat per region, we report some differences in the structure and biodiversity of meiofaunal assemblages comparing both the same habitat from different regions and different habitats within the same region. For instance, in all investigated regions both seagrass and reef sediments generally host the highest number of meiofaunal taxa. These results suggest that, at both investigated spatial scales (i.e., oceanic region and habitat) the differences in meiofaunal diversity appear more evident than those in terms of abundance or biomass.

Although the use of correlations must be always considered with caution, the presence of a significant positive correlation between the number of higher meiofaunal taxa and the number of the nematode expected species number (Fig. 7) suggests that, at least for the investigated regions and habitats, the analysis of biodiversity based on nematodes could be a good proxy for the analysis of the patterns of the whole meiofaunal biodiversity. Such relationship, however, should be further explored on a much larger set of tropical sedimentary environments.

The analysis of structural nematode biodiversity (either expressed in terms of species richness, expected species number, or evenness) is significantly higher in the Coral triangle (Celebes, Indonesia) than in the Caribbean Sea, whatever the considered habitat is. Moreover, although a proper replication of habitats included in our survey would have shed more light on the actual differences among habitats in each region, we report here that in both the Celebes and Caribbean Seas, seagrass beds and reef sediments apparently host a significantly higher species number than mangrove sediments.

A comparative analysis of nematode diversity among different habitats, including reef sediments [60], temperate seagrass beds [61], [62] and mangrove sediments in Australia, Africa, Asia and South America [59], [63]–[65] is difficult as different estimates (indices) of diversity have been used in different studies. Moreover, such a comparison could be biased because of the different geographic locations and sampling efforts [66] as well as by the lack of temporal replication of the surveys. In presence of these potential biases, which altogether could weaken or reinforce the differences among habitats and regions, the results from our study can be nevertheless used at least in comparative terms, as, at all investigated areas, we adopted the same sampling strategy (and sampling effort), sample storage, methodology of extraction and determination/identification procedures.

The comparison of the biodiversity of the different habitats from each region suggests that each single habitat provided an important and significant contribution to the regional (i.e., gamma) nematode diversity only in the Caribbean Sea. In fact, the nematode beta diversity among seagrass, mangrove and reef sediments is >64% in both the Celebes and Caribbean Seas, but the post-hoc tests after the PERMANOVA revealed that the differences in the composition of nematode assemblages were significant only in the Caribbean Sea. This result would suggest that factors regulating nematode gamma diversity in the Caribbean and Celebes Sea are far different each other and leave open this issue to further investigations.

Soft bottoms are typically characterized by environmental variations that operate at the scale of a few centimeters on micro- and meiofaunal assemblages [33]. For instance, a recent study conducted from a single mangrove system from northwestern Brazil reported the existence of significant differences in nematode assemblage structure among micro-habitats [29]. Altogether our results and the evidences available from the literature pinpoint that tropical marine sediments are possibly characterized not only by high levels of nematode biodiversity, but also by high levels of beta diversity at different spatial scales, from the micro-scale to the regional one.

### Relationships between Biodiversity and Ecosystem Functioning

Empirical and theoretical studies increasingly argue that biodiversity regulates the ecosystem functions that are responsible for the production of natural goods and services [2], [67]–[70]. Many investigations relating biodiversity and ecosystem functioning have been performed using controlled field experiments that assemble model (and often non-natural) communities to measure the effects of changes in diversity on several ecosystem processes. Meta-analyses have also shown that species diversity generally has a positive effect on ecosystem processes and that this effect is remarkably consistent across trophic levels and different ecosystems [11]–[12], [70].

In this study, the relationships between the richness of meiofaunal taxa and the nematodes species diversity vs. prokaryotic heterotrophic production are positive linear across all investigated regions and habitats (Fig. 8A–B). Our results are slightly different from those recently reported from coral reefs, where using fish as a model for biodiversity, the relationship appears to be positive exponential [71]. Nevertheless, this allows us to hypothesize that positive relationships could be a peculiar characteristic of tropical shallow habitats, but underpin also that the shape of the relationship (linear vs. exponential) could vary when different components of biodiversity (e.g., meiofauna, nematodes and fish) are taken into account. Differences in the shape of the relationships between biodiversity and ecosystem functioning from different marine environments also emerge when comparing habitats from different water depth. For instance, a positive exponential relationship has been observed in the deep Atlantic, Pacific and Antarctic Oceans and in the deep Mediterranean Sea (at depths ranging 200–8200 m) [18], whereas a recent study conducted along the upper slope off New Zealand (at depths ranging 264–1238 m) reported linear negative or null relationships [19]. Moreover, a recent manipulative experiment carried out on natural nematode assemblages response to thermal stress [72] led to hypothesize the same probability of (saturating) rivet-like [73] or idiosyncratic [74] relationships between nematode species richness and ecosystem functioning.

The large variability in the shape of the relationships between marine biodiversity and ecosystem functioning can be due to: i) the relatively limited number of regions and habitats considered so far, ii) the different approaches used (i.e., correlative from the real world and manipulative experiments in the laboratory), and iii) the different environmental characteristics of the investigated regions and habitats (e.g., coastal vs. deep-sea sediments). These discrepancies do not allow us making any robust speculation about the possible mechanisms explaining the different shapes in the relationship between biodiversity and ecosystem functioning observed in different marine ecosystems. Nevertheless, our results corroborate the presence of a pre-eminently positive effect of biodiversity on marine ecosystem functioning, and let us concluding that any loss in marine metazoan biodiversity (whatever the phylum considered) could result in a variably severe impairment of marine ecosystem functions.

Finally, it must be taken into account that the correlative approach used in this and other previous studies leaves open yet the conceptual possibility to interpret the reverse relationship, i.e. addressing whether and how ecosystem functions control biodiversity. This was not among our aims, but we must acknowledge that, in our study, the reverse relationship would remain positive linear and this would confirm that meiofaunal (and nematode) biodiversity are tightly dependent on the functioning of the microbial loop [26].

## Supporting Information

Figure S1 **Contribution of all taxa (i.e., nematodes, copepods and polychaetes excluded) to the meiofaunal communities in the seagrass, mangrove and reef habitat of the Caribbean Sea (A), Red Sea (B) and Celebes Sea (C).**
(DOCX)

Table S1 **Meiofaunal and nematode beta diversity among habitats within each region and among regions for each habitat separately.**
(DOCX)
