# Supplementary material for: Relationships between Meiofaunal Biodiversity and Prokaryotic Heterotrophic Production in Different Tropical Habitats and Oceanic Regions
Source: PLoS One. 2014 Mar 6;9(3):e91056. doi: 10.1371/journal.pone.0091056 (PMC3948168; doi:10.1371/journal.pone.0091056)
Supplement: Table S1 — Meiofaunal and nematode beta diversity among habitats within each region and among regions for each habitat separately. (DOCX) [file pone.0091056.s002.docx]

| **All meiofaunal taxa** | **Habitat type** | **Average dissimilarity (%)** |  |  | **Region** | **Average dissimilarity (%)** |
| --- | --- | --- | --- | --- | --- | --- |
| **Caribbean Sea** | **seagrass vs. mangrove** | 60 |  | **seagrass** | **Caribbean vs. Red** | 28 |
|  | **mangrove vs. reef** | 52 |  |  | **Caribbean vs. Celebes** | 27 |
|  | **seagrass vs. reef** | 37 |  |  | **Red vs. Celebes** | 30 |
|  |  |  |  |  |  |  |
| **Red Sea** | **seagrass vs. mangrove** | 41 |  | **mangrove** | **Caribbean vs. Red** | 53 |
|  | **mangrove vs. reef** | 30 |  |  | **Caribbean vs. Celebes** | 61 |
|  | **seagrass vs. reef** | 44 |  |  | **Red vs. Celebes** | 44 |
|  |  |  |  |  |  |  |
| **Celebes Sea** | **seagrass vs. mangrove** | 26 |  | **reef** | **Caribbean vs. Red** | 42 |
|  | **mangrove vs. reef** | 29 |  |  | **Caribbean vs. Celebes** | 29 |
|  | **seagrass vs. reef** | 17 |  |  | **Red vs. Celebes** | 48 |
|  |  |  |  |  |  |  |
| **nematode diversity** |  |  |  |  |  |  |
| **Caribbean Sea** | **seagrass vs. mangrove** | 74 |  | **seagrass** | **Caribbean vs. Celebes** | 68 |
|  | **mangrove vs. reef** | 93 |  |  |  |  |
|  | **seagrass vs. reef** | 83 |  |  |  |  |
|  |  |  |  | **mangrove** | **Caribbean vs. Celebes** | 77 |
|  |  |  |  |  |  |  |
| **Celebes Sea** | **seagrass vs. mangrove** | 71 |  |  |  |  |
|  | **mangrove vs. reef** | 79 |  | **reef** | **Caribbean vs. Celebes** | 83 |
|  | **seagrass vs. reef** | 64 |  |  |  |  |
