# Supplementary material for: Relationships between Meiofaunal Biodiversity and Prokaryotic Heterotrophic Production in Different Tropical Habitats and Oceanic Regions
Source: PLoS One. 2014 Mar 6;9(3):e91056. doi: 10.1371/journal.pone.0091056 (PMC3948168; doi:10.1371/journal.pone.0091056)
Supplement: Supplementary file 2 [file pone.0091056.s001.docx]

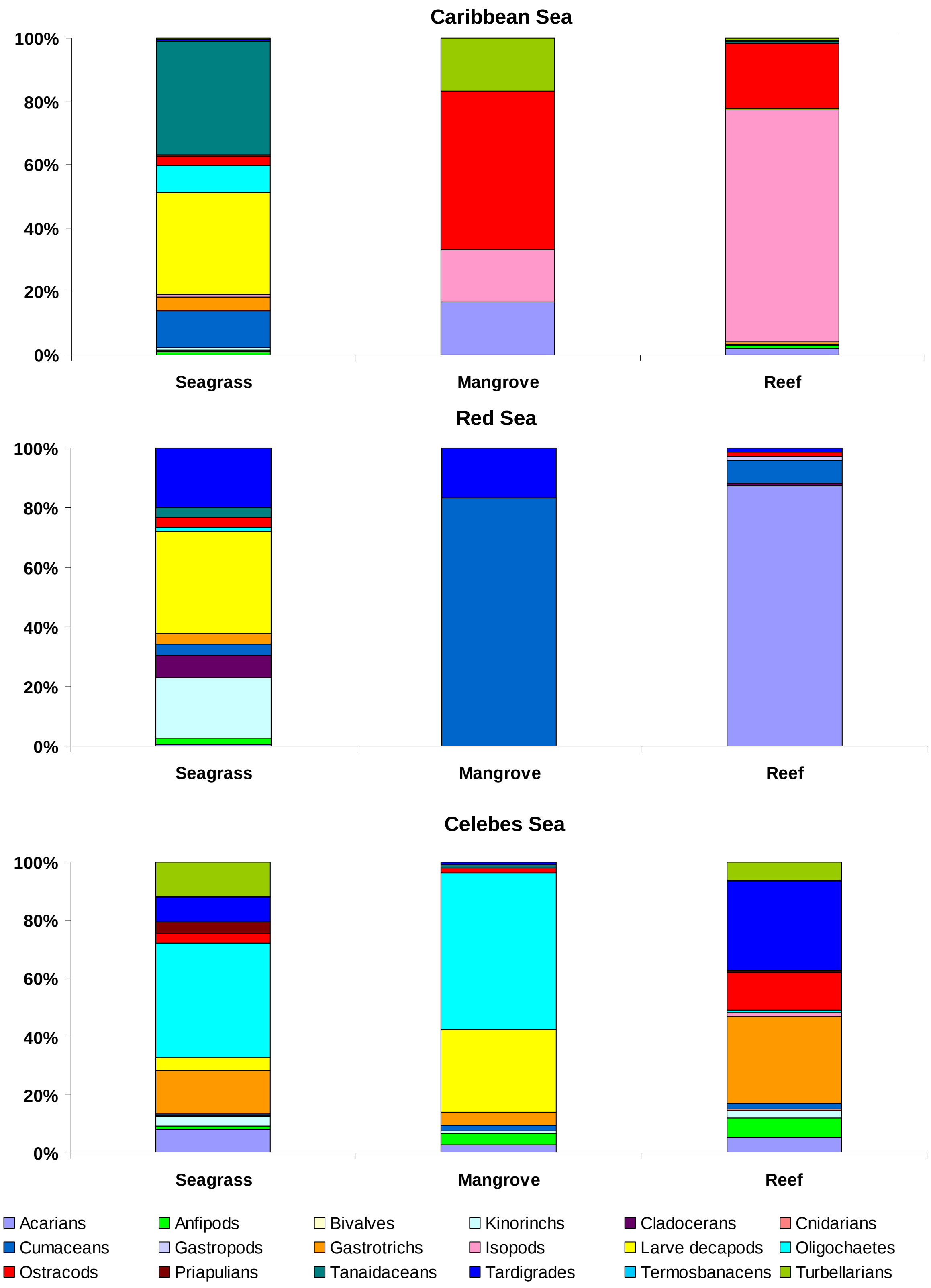
Caribbean Sea
100%
80%
60%
40%
20%
0%
Seagrass
Mangrove
Reef
Red Sea
100%
80%
60%
40%
20%
0%
Seagrass
Mangrove
Reef
Celebes Sea
100%
80%
60%
40%
20%
0%
Seagrass
Mangrove
Reef
Acarians
Anfipods
Bivalves
Kinorinchs
Cladocerans
Cnidarians
Cumaceans
Gastropods
Gastrotrichs
Isopods
Larve decapods
Oligochaetes
Ostracods
Priapulians
Tanaidaceans
Tardigrades
Termosbanacens
Turbellarians
